# An Atypical Presentation of Hemophagocytic Lymphohistiocytosis (HLH) Secondary to Occult Hodgkin Lymphoma

**DOI:** 10.1155/2021/6672257

**Published:** 2021-07-24

**Authors:** Justin Komisarof, Kevin McGann, Alissa Huston, Hani Katerji, Mary Anne Morgan

**Affiliations:** ^1^Department of Medicine, University of Rochester Medical Center, 601 Elmwood Avenue Box MED, Rochester, NY 14642, USA; ^2^Department of Pathology and Laboratory Medicine, University of Rochester Medical Center, 601 Elmwood Avenue Box 626, Rochester, NY 14642, USA

## Abstract

Hemophagocytic lymphohistiocytosis (HLH) is a rare and life-threatening syndrome of immune system dysregulation characterized by the phagocytosis of various cells by histiocytes in the bone marrow. HLH can present in one of the two ways: primary HLH, which is caused by mutations in genes essential to T and NK-cell function, and secondary HLH, typically caused by Epstein–Barr virus (EBV) infection or malignancy. Because of the rapid progression and high mortality of this disease, prompt diagnosis is essential to good outcomes. Here, we report the 2-month clinical course of a patient who presented with altered mental status and recurrent fever of unknown origin. Initially, he did not meet diagnostic criteria for HLH and had a negative bone marrow biopsy; however, he eventually progressed to full-blown HLH secondary to occult Hodgkin lymphoma. This case is unusual for the slow and smoldering course of the patient's disease and highlights the importance of aggressively searching for potential malignancies to ensure the initiation of definitive therapy as soon as possible.

## 1. Introduction

Hemophagocytic lymphohistiocytosis (HLH) is a rare life-threatening syndrome of immune system dysregulation characterized by hypersecretion of inflammatory cytokines due to persistent overactivation of cytotoxic T cells and NK cells, ultimately leading to phagocytosis of hematopoietic cells by histiocytes throughout the reticuloendothelial system [1–3]. While HLH was initially described as a genetic disorder of childhood and much of what is known regarding its pathophysiology, presentation, and management has originated from the pediatric literature, our understanding of secondary HLH in adults continues to grow [4]. While the potential etiologic underpinnings of secondary HLH are exceedingly broad [5–10], principal among them are EBV infection and lymphoma [1, 11–14]. The presentation of HLH is variable and nonspecific, overlapping with many conditions such as sepsis, thereby posing significant challenges for early diagnosis and treatment in what is often a rapidly progressive and fatal picture [15–21].

Diagnostic criteria developed from pediatric patients with familial HLH were first proposed in 1991 [22] and later revised in 2004 [23] to include eight criteria of which a patient must meet at least five: fever (≥38.5°C for ≥7 days), cytopenias (≥2 cell lines affected), splenomegaly (>3 cm below costal margin), hypertriglyceridemia (≥2 mmol/L) and/or hypofibrinogenemia (≤150 mg/dL), hemophagocytosis in the bone marrow, spleen, or lymph nodes, elevated ferritin (≥500 *μ*g/l), elevated soluble IL-2 receptor levels (≥2400 U/mol), and low/absent NK-cell activity. More recently, novel diagnostic criteria known as the HScore have also been developed and validated in a small population of adult patients [24]. These diagnostic criteria align with the most common presenting symptoms of HLH and include fever, splenomegaly, and cytopenias. However, an array of other presenting findings, including skin rash [3, 25, 26], pulmonary symptoms such as dyspnea and cough [27], and neurologic features including encephalopathy, seizure, ataxia, and cranial nerve abnormalities have been reported [28–31]. Treatment of secondary HLH must be initiated early and aims to address the underlying precipitating disorder, with the most commonly used HLH protocol relying on etoposide, dexamethasone, and hematopoietic stem cell transplantation as mainstays of therapy [32]. For HLH secondary to Hodgkin lymphoma, brentuximab therapy has been reported to be successful [33, 34].

## 2. Case Presentation

**Figure 1:**
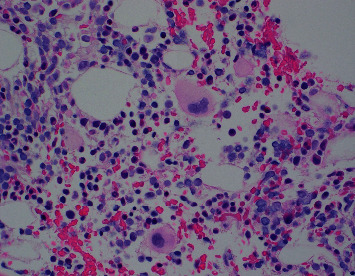
Hematoxylin and eosin (H&E) section of the hypocellular bone marrow with no definitive infiltration by lymphoma.

A 63-year-old man with a history of remote Hodgkin lymphoma at age 19 years, coronary artery disease (CAD), and recent urothelial carcinoma presented to Highland Hospital with 2-3 months of intermittent fevers and confusion. His initial laboratory values were notable for anemia (hemoglobin 9.0 g/dL) and thrombocytopenia (63,000 platelets/*μ*L) with a normal white blood cell count (5700 WBC/*μ*L). He was hyponatremic with a sodium of 125 mmol/L and had an elevated alkaline phosphatase (300 U/L). He underwent an extensive infectious workup which was notable only for positive Epstein–Barr virus (EBV) viral PCR (7800 copies/mL) but negative EBV IgM. Hematology was consulted out of concern for a hemolytic anemia, but hemolysis labs were unremarkable with normal serum haptoglobin (93 mg/dL) and total bilirubin (0.9 mg/dL). Reticulocyte count was not elevated (2.3%), and schistocytes were not seen on a peripheral smear. The possibility of hemophagocytic lymphohistiocytosis (HLH) was raised at this time due to the patient's cytopenias and recurrent fever. At this time, his ferritin was elevated at 1089 ng/mL and triglycerides were elevated at 153 mg/dL but below the diagnostic cutoff for HLH. However, fibrinogen was normal, and the patient was not observed to have splenomegaly. A bone marrow biopsy was performed and did not show phagocytic histiocytes or evidence of malignancy (Figure 1). NK functional assay was sent but cancelled due to insufficient lymphocyte count. Soluble IL-2 receptor assay was ordered. The patient was treated with a 5-day course of antibiotics, and his fever curve gradually downtrended. His encephalopathy improved with the resolution of his fevers. The patient was afebrile for 72 hours and was able to be discharged from the hospital.

**Figure 2:**
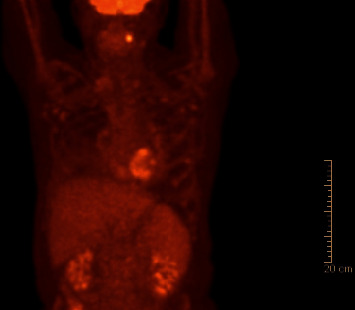
PET scan revealing the hypermetabolic focus localized to the left palatine tonsil.

**Figure 3:**
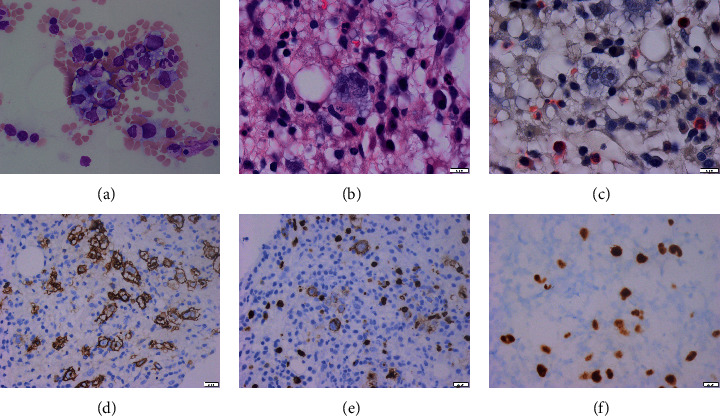
H&E section of the bone marrow revealing hemophagocytosis (a). High-powered H&E section (b) and chloracetate esterase section (c) demonstrating binucleated Reed–Sternberg (RS) cells. Immunohistochemistry showed positivity for CD30 (d) and CD15 (e) in RS cells. Epstein–Barr encoding region in situ hybridization (EBER-ISH) (f).

One month later, the patient was admitted to Strong Memorial Hospital with ongoing intermittent fevers and confusion. In the interim, he underwent a positron emission tomography (PET) scan which revealed a hypermetabolic focus in the left palatine tonsil (Figure 2). On admission, triglycerides were slightly higher at 173 mg/dL and ferritin was also slightly higher at 1407 ng/mL. He had a positive blood culture for *Staphylococcus epidermidis* which was believed to be a contaminant. Tonsillar biopsy demonstrated an atypical clonal lymphoid population but no morphologically abnormal lymphoid cells and no Hodgkin Reed–Sternberg (HRS) cells or other evidence for lymphoma. Soluble IL-2 receptor assay from the earlier hospitalization had still not resulted at this time. Ferritin continued to rise climbing steadily to 9573 ng/mL two weeks after admission. The patient became hypotensive and was transferred to the ICU for vasopressor support. He developed acute hypoxic respiratory failure requiring intubation thought to be due to pleural effusions and atelectasis. Soluble IL-2 receptor came back extremely elevated at 17,375 U/mL. Repeat EBV viral PCR was now 40,400 copies/mL. The patient underwent repeat bone marrow biopsy which showed Hodgkin lymphoma with hemophagocytic lymphohistiocytosis (Figure 3), confirming the diagnosis of HLH. Karyotype analysis was 46XY with no apparent clonal chromosomal aberrations, and EBER-ISH was positive. The patient was started on high-dose dexamethasone (20 mg) daily as well as etoposide (100 mg/mL) twice weekly. At this point, ferritin was now 12,130 ng/mL. Dexamethasone was increased shortly after to 40 mg daily, and the patient received a single dose of brentuximab (1.8 mg/kg). After receiving brentuximab, the patient developed progressive renal failure with creatinine increasing from 1.4 to 3.0 mg/dL over the course of a week as well as a lactic acidosis. After discussion with the family, the patient was terminally extubated and passed away.

## 3. Discussion

Hemophagocytic lymphohistiocytosis (HLH) is a rare life-threatening syndrome of immune system dysregulation typically seen secondary to EBV infection or blood malignancy. Herein, we describe the detailed 2-month course of a 63-year-old male who presented with altered mental status, recurrent fevers, and fatigue and who was ultimately found to have HLH secondary to occult Hodgkin lymphoma.

This case is unusual for its slow and smoldering presentation. Typically, patients with active HLH present acutely ill, often in multiple organ system failure and requiring ICU level of care. This patient's symptoms fluctuated to an extent that he met criteria for hospital discharge, only to be readmitted four weeks later with ongoing subacute and intermittent symptoms. Although the diagnosis of HLH secondary to new lymphoma was entertained during the patient's first hospital stay, the bone marrow biopsy was unrevealing. During his second hospital stay, despite multidisciplinary subspecialty consultation and interdepartmental review, the diagnosis was clinched only when the bone marrow aspirate was repeated. As no malignant cells were found outside the bone marrow, it is possible that this represented primary bone marrow Hodgkin lymphoma. While this is quite rare and typically associated with HIV infection, for which our patient tested negative, several previous cases have been described of HIV-negative primary bone marrow Hodgkin lymphoma [35, 36]. Alternatively, it is possible that the tonsillar biopsy was a false negative and that this represented stage 4 Hodgkin lymphoma.

The most notable feature of this case that delayed diagnosis was the first bone marrow biopsy which did not show any evidence of phagocytic histiocytes or underlying malignancy. While HLH is characterized by hemophagocytosis, it is important to note that the sensitivity of bone marrow biopsy for HLH is relatively low at 83% and that hemophagocytosis can be seen in the bone marrow of patients without HLH [37]. Similarly, it is becoming clear that bone marrow biopsy false negatives are fairly common in Hodgkin lymphoma and that patients should undergo PET/CT as part of their initial workup [38]. HLH is treated primarily with a regimen of dexamethasone and etoposide, and HLH secondary to malignancy must be treated by addressing the underlying cancer [39]. In all cases where HLH is suspected, an aggressive approach must be taken both to work up all HLH diagnostic criteria as well as to identify any underlying malignancy present and initiate definitive treatment as quickly as possible.

## Data Availability

The imaging, laboratory, and pathologic data used to support the findings of this case study are included within the article.
